# Change in life expectancy with type 2 diabetes: a study using claims data from lower Saxony, Germany

**DOI:** 10.1186/s12963-017-0124-6

**Published:** 2017-02-13

**Authors:** Denise Muschik, Juliane Tetzlaff, Karin Lange, Jelena Epping, Sveja Eberhard, Siegfried Geyer

**Affiliations:** 1 Medical Sociology Unit, Hannover Medical School, Carl-Neuberg-Str.1, 30625 Hannover, Germany; 2 Medical Psychology Unit, Hannover Medical School, Carl-Neuberg-Str.1, 30625 Hannover, Germany; 3 AOK Niedersachsen – Statutory Health Insurance of Lower Saxony, Hannover, Germany

**Keywords:** Type 2 diabetes, Life expectancy, Expansion of morbidity, Dynamic equilibrium, Compression of morbidity

## Abstract

**Background:**

This study estimates life expectancy with and without type 2 diabetes for individuals in Lower Saxony, Germany in order to detect a trend in population health.

**Methods:**

Morbidity and mortality data derived from German administrative claims data (statutory health insurance, AOK Niedersachsen, *N* = 2,900,065) were used covering 10 years from 2005 to 2014. Life table analysis was applied for calculating life expectancy, life expectancy free of type 2 diabetes, life expectancy with type 2 diabetes, and the proportion of life expectancy free of diabetes to total life expectancy using the Sullivan method.

**Results:**

The total life expectancy increase is stronger in men than in women: At the age of 20, total life expectancy was 55.0 years in 2005 and 56.3 years in 2014 for men, whereas it was 61.7 years in 2005 and 62.5 years in 2014 for women. Decreases in life expectancy without type 2 diabetes were more pronounced in women than in men. Accordingly, life expectancy with type 2 diabetes increased in both women and in men. The proportion of life expectancy without diabetes to total life expectancy decreased, indicating a similar development in both. For example, at the age of 60, the proportion of life expectancy without diabetes to total life expectancy decreased from 0.75 in 2005 to 0.66 in 2014 for men, while it decreased from 0.77 in 2005 to 0.70 in 2014 for women.

**Conclusions:**

Against the background of increasing total life expectancy, the time spent in morbidity increased for the case of type 2 diabetes in Lower Saxony, Germany.

## Background

Population aging leads to an increase in chronic diseases, with type 2 diabetes being one of the most relevant issues [1]. From 1980 to 2014, the global prevalence of diabetes in adults almost doubled because of an increase in type 2 diabetes [2]. Tamayo and colleagues [3] estimated the number of Europeans with diabetes between 20 and 79 years to be 56 million in 2013. This estimate corresponds to a raw overall prevalence of 8.5% and an age-standardized prevalence of 6.8%. An increase of nearly 10 million people with diabetes is predicted for 2035.

As life expectancy is increasing [4], whether the additional life year gains are spent in good or in poor health must be examined. Three hypotheses have been formulated to outline the ways morbidity might develop. Fries [5] assumed that the onset of impairment and morbidity would be postponed to a higher age, which might proceed at a faster pace than the increase of life expectancy, therefore leading to shorter time periods spent in poor health. Fries called this concept *compression of morbidity* and attributed it to successful primary prevention and improved living conditions. A different hypothesis was proposed by Gruenberg [6], who assumed that medical progress prolongs life with chronic diseases, thus leading to *morbidity expansion*. Finally, Manton [7] combined both perspectives by assuming that, even with increases in life expectancy, the relative number of life years spent in good and poor health may remain unchanged, and that, due to medical progress, the quality of life would be improved. Manton called this a *dynamic equilibrium*.

As longitudinal data are rare, few studies on these concepts have been conducted thus far. Depending on the country, the data, and the outcomes measured, findings on changing morbidity patterns vary but point in the same direction for type 2 diabetes. Prevalence is reported to increase [1, 2], and onset age is shifted into younger age groups, mainly due to obesity in childhood and adolescence [8–11]. The corresponding development of morbidity with type 2 diabetes may thus be described as expansion. Furthermore, Loukine and colleagues [12] reported a lower total life expectancy and a lower health-adjusted life expectancy in patients with type 2 diabetes than in individuals without the chronic condition. Tancredi and colleagues [13] analyzed Swedish registry data and found higher mortality in type 2 diabetes patients than in the general population.

The use of registry or claims data alone or in combination with other data sources is growing in diabetes research [14]: based on the data of several German health insurance providers, Hoffmann and Icks [15] reported an overall prevalence of diabetes of 6.9% in individuals between 18 and 79 years. However, regional differences in Germany have to be considered: reported age-standardized incidence rates of type 2 diabetes ranged from 0.9% to 1.7% [16]. Due to the heterogeneous results, more research on the development of type 2 diabetes is needed. Many studies covered a limited age span or were based on low case numbers. The aim of our study was to investigate the development of life expectancy free of type 2 diabetes and life expectancy with type 2 diabetes over time by using a large claims database covering 10 years. Whether life expectancy with type 2 diabetes increased over a longer observation period requires examination. Our hypothesis was that life expectancy with type 2 diabetes may increase over time. Compression of morbidity would be present if total and life expectancy free of type 2 diabetes had increased, and if life expectancy with type 2 diabetes had decreased in relative terms.

To our knowledge, this is the first German study using health insurance data for estimating life expectancy free of type 2 diabetes and life expectancy with type 2 diabetes. The advantage to using this database is its completeness. All diagnosed cases of the insurance population are included, and data are also available if subjects were temporarily or permanently unable or reluctant to respond.

In Germany, a national disease management program for type 2 diabetes was introduced in 2002 within the framework of the German Social Code, Book V (SGB V) [17, 18]. This program is available for all patients with statutory health insurance, which applies to the vast majority of the population. The disease management program defines a quality assured course of treatment reflecting the current national clinical guidelines for the treatment of type 2 diabetes.

## Methods

### Data

The database consists of anonymized statutory health insurance claims data from Lower Saxony, Germany (Allgemeine Ortskrankenkasse Niedersachsen). For the years 2005 to 2014, data for *N* = 2,900,065 insured are available, and for each year, approximately two million women and men with a balanced gender ratio are included. Originally, the data were collected for accounting purposes. They contain demographic variables, inpatient and outpatient diagnoses (coded according to ICD-10), as well as outpatient medications coded according to ATC [19]. The lower age limit for inclusion is 18 years. As health insurance providers maintain their records only for a limited time period, data are available for 2005 to 2014 only.

### Statistical analyses

Age-standardized prevalence and mortality rates were calculated for all calendar years. A comparison between the different years is possible by keeping the age structure constant over time. This was achieved by using the age distributions of men and women in 2009 as the standard population.

Total life expectancy, life expectancy free of type 2 diabetes, life expectancy with type 2 diabetes, and the proportion of life expectancy without diabetes to total life expectancy were estimated. First, we applied a period life table analysis based on our dataset to calculate the total life expectancy [20]. This is the basis for the Sullivan method [21] that we applied next, and which is often used in demographic analyses to assess the health status of a population. The Sullivan method combines information on morbidity and mortality to estimate years lived without and with a specific disease. In our case, age-specific type 2 diabetes prevalence rates were linked to the period life table. The Sullivan method enables the division of total life expectancy into life expectancy without type 2 diabetes and life expectancy with type 2 diabetes. Thus, the expected average life years without and with type 2 diabetes at a specific age can be determined. Additionally, the proportion of life expectancy without diabetes to total life expectancy was computed with this method, depicting the relative amount of expected healthy years compared to total life expectancy for each year of age. We generated period life tables for each year from 2005 to 2014 separately for men and women in order to obtain the total life expectancy. As the data were available from age 18, the life table began at this age and ended with the final age group of 100+. We used the unabridged life table because data were available for each single year of age. The claims data contain information on the year of birth and the year of death as well as the duration of insurance period. Based on the number of days an individual was insured in a respective year, the mid-year population was calculated. Age-specific mortality rates were obtained by dividing the total deaths by the mid-year population for every single age. There is no information on the cause of death. Age-specific type 2 diabetes prevalence rates were calculated in the same way. The different life expectancies were displayed starting from age 20 in 10-year intervals up to 80 years.

### Classification of type 2 diabetes cases

The identification of individuals with type 2 diabetes from health insurance data is methodologically challenging. Due to the occurrence of coding errors, different diabetes diagnoses might appear for one individual over the entire insurance period. However, we can limit the error because 80 to 90% of all diabetes cases can be attributed to type 2 [22].

Our goal was to minimize this source of error. Therefore, type 2 diabetes patients were selected by applying a stepwise procedure: outpatient and inpatient physician diagnosis codes according to ICD-10 are available quarterly. We classified individuals as having type 2 diabetes if they predominantly received ICD-10 E11 diagnoses (out of all appearing diabetes diagnoses) in their insurance period. We decided to include men and women who most frequently had the unspecific diagnosis ICD-10 E14, as the majority of diabetes patients have type 2. Furthermore, prescribed diabetes medications coded according to the ATC-classification were available. This enabled the identification of individuals receiving oral antidiabetics (ATC-A10B) or insulin (ATC-A10A). As not all type 2 diabetes patients are treated with medication, this could not be the only method for validation. If men or women predominantly had type 1 diabetes diagnoses (among other diabetes diagnoses), but did not have an insulin prescription in the entire insurance period, we classified them as implausible cases of type 1 diabetes. Therefore, they were assigned to the group of type 2 diabetes patients. Individuals with a diabetes diagnosis in only one quarter without any medication appearing in the entire insurance period but who were insured for longer than one quarter were defined as implausible cases and therefore excluded. Gaps without a diabetes diagnosis could appear in one individual over the insurance period between 2005 and 2014. In these cases, we still classified the individual as having type 2 diabetes because the disease is chronic.

Data management and the analyses were performed in Stata 11MP [23] and R [24].

## Results

### Prevalence

Age-standardized prevalence rates of type 2 diabetes increased from 2005 to 2014 (Table 1). However, they were generally higher in women than in men. Age-standardized prevalence rates in men rose from 10.7% in 2005 to 14.5% in 2014. In women, the rate was 12.0% in 2005 and 15.8% in 2014. The total number of insured individuals as well as the number of deaths remained similar in women, but in men, the number of deaths increased somewhat over the ten-year period. Age-standardized mortality rates declined slightly over the observation period in women and in men.

**Table 1:** Type 2 diabetes cases, age-standardized prevalence rates of type 2 diabetes^a^, number of deaths, age-standardized mortality rates^a^ and number of all insured (N) by year and sex

	Year	Type 2 diabetes cases	Age-standardized prevalence rates of type 2 diabetes	Deaths	Age-standardized mortality rates	N
Men	2005	89 121	10.7%	14 404	1.8%	900 551
	2006	97 107	11.7%	14 360	1.8%	894 701
	2007	102 279	12.3%	14 254	1.8%	888 370
	2008	107 113	12.9%	14 512	1.8%	874 690
	2009	110 682	13.3%	14 618	1.8%	875 488
	2010	127 553	13.8%	15 645	1.7%	1 012 304
	2011	132 124	13.8%	15 954	1.7%	1 020 440
	2012	134 949	14.0%	16 208	1.6%	1 025 982
	2013	135 129	14.0%	16 781	1.7%	1 026 358
	2014	140 232	14.5%	16 345	1.6%	1 032 109
	Total	1 176 289		153 081		9 550 993
Women	2005	119 074	12.0%	19 376	2.0%	1 041 281
	2006	126 766	12.9%	18 645	1.9%	1 030 198
	2007	131 660	13.5%	18 454	1.9%	1 018 332
	2008	136 148	14.1%	18 717	1.9%	999 761
	2009	138 651	14.5%	18 570	1.9%	993 029
	2010	152 752	15.0%	19 374	1.9%	1 103 039
	2011	156 454	15.1%	18 897	1.8%	1 104 183
	2012	158 531	15.3%	19 255	1.9%	1 102 032
	2013	157 316	15.4%	19 806	1.9%	1 094 618
	2014	161 701	15.8%	18 614	1.8%	1 092 462
	Total	1 439 053		189 708		10 578 935

^a^Standard population AOK NDS 2009

### Total life expectancy

Total life expectancy increased slightly from 2005 to 2014 in women and in men (Table 2). For example, the total life expectancy of women at the age of 20 amounted to 61.7 years in 2005 and 62.5 years in 2014. In men at the age of 20, the total life expectancy was 55.0 years in 2005 and 56.3 years in 2014. At age 80, men were expected to live 7.0 years in 2005 and 7.6 years in 2014. Women at age 80 had a total life expectancy of 8.8 years in 2005 and 9.5 years in 2014. Regardless of the selected age or year, women always showed higher values than men. Comparing 2014 to 2005, the increases were significant for all ages and both sexes.

**Table 2:** Total life expectancy and confidence intervals (95%) by year and sex at selected ages

	Age	2005	2006	2007	2008	2009	2010	2011	2012	2013	2014
Men	20	55.0	55.1	55.1	55.2	55.2	55.7	56.0	56.2	56.0	56.3
		(54.8 – 55.2)	(54.9 – 55.3)	(54.9 – 55.3)	(55.0 – 55.4)	(55.0 – 55.5)	(55.5 – 55.9)	(55.8 – 56.2)	(56.0 – 56.4)	(55.8 – 56.2)	(56.1 – 56.5)
	30	45.4	45.5	45.6	45.6	45.6	46.1	46.3	46.5	46.3	46.6
		(45.2 – 45.6)	(45.3 – 45.7)	(45.4 – 45.8)	(45.4 – 45.9)	(45.4 – 45.8)	(45.8 – 46.3)	(46.1 – 46.5)	(46.3 – 46.7)	(46.1 – 46.5)	(46.4 – 46.8)
	40	35.9	36.1	36.1	36.1	36.1	36.5	36.8	36.9	36.7	37.1
		(35.7 – 36.1)	(35.9 – 36.3)	(35.9 – 36.3)	(35.9 – 36.3)	(35.9 – 36.3)	(36.3 – 36.7)	(36.6 – 37.0)	(36.7 – 37.1)	(36.5 – 36.9)	(36.9 – 37.3)
	50	27.0	27.1	27.3	27.2	27.3	27.5	27.7	27.8	27.6	28.0
		(26.8 – 27.2)	(26.9 – 27.3)	(27.1 – 27.5)	(27.0 – 27.4)	(27.1 – 27.5)	(27.3 – 27.7)	(27.6 – 27.9)	(27.6 – 28.0)	(27.4 – 27.8)	(27.8 – 28.1)
	60	19.2	19.2	19.3	19.3	19.5	19.6	19.8	19.7	19.7	19.9
		(19.0 – 19.3)	(19.1 – 19.4)	(19.2 – 19.5)	(19.1 – 19.4)	(19.3 – 19.7)	(19.5 – 19.8)	(19.7 – 20.0)	(19.6 – 19.9)	(19.5 – 19.8)	(19.7 – 20.0)
	70	12.4	12.5	12.7	12.7	12.7	12.9	13.1	13.0	12.9	13.2
		(12.3 – 12.5)	(12.4 – 12.7)	(12.5 – 12.8)	(12.6 – 12.8)	(12.6 – 12.9)	(12.7 – 13.0)	(13.0 – 13.2)	(12.9 – 13.1)	(12.8 – 13.0)	(13.1 – 13.3)
	80	7.0	7.1	7.4	7.2	7.2	7.4	7.5	7.4	7.3	7.6
		(6.9 – 7.2)	(7.0 – 7.3)	(7.2 – 7.5)	(7.1 – 7.3)	(7.1 – 7.3)	(7.3 – 7.5)	(7.4 – 7.6)	(7.3 – 7.6)	(7.2 – 7.4)	(7.5 – 7.7)
Women	20	61.7	61.9	61.9	61.8	61.7	62.0	62.3	62.3	62.0	62.5
		(61.5 – 61.9)	(61.7 – 62.1)	(61.7 – 62.1)	(61.6 – 62.0)	(61.5 – 61.9)	(61.8 – 62.1)	(62.1 – 62.5)	(62.1 – 62.5)	(61.8 – 62.2)	(62.3 – 62.7)
	30	51.9	52.0	52.1	52.0	52.0	52.2	52.5	52.4	52.2	52.6
		(51.7 – 52.0)	(51.8 – 52.2)	(52.0 – 52.3)	(51.8 – 52.2)	(51.8 – 52.2)	(52.0 – 52.4)	(52.3 – 52.6)	(52.2 – 52.6)	(52.0 – 52.3)	(52.4 – 52.8)
	40	42.2	42.2	42.4	42.3	42.3	42.5	42.7	42.7	42.5	42.9
		(42.0 – 42.3)	(42.1 – 42.4)	(42.2 – 42.6)	(42.1 – 42.5)	(42.1 – 42.5)	(42.3 – 42.6)	(42.6 – 42.9)	(42.5 – 42.9)	(42.3 – 42.7)	(42.7 – 43.0)
	50	32.9	32.9	33.1	33.0	32.9	33.0	33.3	33.3	33.0	33.4
		(32.7 – 33.0)	(32.8 – 33.1)	(32.9 – 33.3)	(32.8 – 33.2)	(32.8 – 33.1)	(32.9 – 33.2)	(33.2 – 33.5)	(33.1 – 33.5)	(32.9 – 33.2)	(33.3 – 33.6)
	60	24.0	24.3	24.3	24.2	24.2	24.3	24.5	24.5	24.3	24.7
		(23.9 – 24.2)	(24.1 – 24.4)	(24.2 – 24.5)	(24.1 – 24.4)	(24.1 – 24.4)	(24.1 – 24.4)	(24.4 – 24.7)	(24.4 – 24.7)	(24.2 – 24.5)	(24.5 – 24.8)
	70	15.9	16.1	16.2	16.1	16.1	16.2	16.5	16.4	16.3	16.7
		(15.8 – 16.0)	(15.9 – 16.2)	(16.0 – 16.3)	(16.0 – 16.3)	(16.0 – 16.3)	(16.1 – 16.3)	(16.4 – 16.6)	(16.3 – 16.5)	(16.2 – 16.4)	(16.6 – 16.9)
	80	8.8	8.9	9.0	8.9	9.0	9.0	9.3	9.2	9.0	9.5
		(8.7 – 8.9)	(8.8 – 9.0)	(9.0 – 9.1)	(8.8 – 9.0)	(8.9 – 9.1)	(8.9 – 9.1)	(9.2 – 9.4)	(9.1 – 9.3)	(8.9 – 9.1)	(9.4 – 9.6)

### Life expectancy without type 2 diabetes

Life expectancy without type 2 diabetes decreased continuously in women and in men but was more pronounced in women (Table 3). Analogously to total life expectancy, female life expectancy without type 2 diabetes was higher than that of males. For example, in males, life expectancy without type 2 diabetes at age 60 was 14.4 years in 2005 and 13.1 years in 2014, whereas it amounted to 18.5 years in 2005 and 17.2 years in 2014 for women at age 60. Again, regardless of the selected age or year, women were always expected to live more years without type 2 diabetes than men. The decreases were significant for all ages and both sexes when comparing 2014 to 2005.

**Table 3:** Life expectancy without type 2 diabetes and confidence intervals (95%) by year and sex at selected ages

	Age	2005	2006	2007	2008	2009	2010	2011	2012	2013	2014
Men	20	49.3	48.9	48.5	48.3	48.1	48.2	48.4	48.4	48.3	48.3
		(49.2 – 49.5)	(48.7 – 49.1)	(48.4 – 48.7)	(48.2 – 48.5)	(48.0 – 48.3)	(48.0 – 48.3)	(48.2 – 48.5)	(48.3 – 48.6)	(48.1 – 48.4)	(48.1 – 48.4)
	30	39.7	39.3	39	38.7	38.5	38.5	38.7	38.7	38.6	38.5
		(39.5 – 39.9)	(39.1 – 39.5)	(38.8 – 39.2)	(38.6 – 38.9)	(38.3 – 38.6)	(38.4 – 38.6)	(38.5 – 38.8)	(38.6 – 38.8)	(38.4 – 38.7)	(38.4 – 38.7)
	40	30.2	29.9	29.6	29.3	29.1	29	29.2	29.2	29.1	29.1
		(30.1 – 30.4)	(29.7 – 30.0)	(29.5 – 29.8)	(29.1 – 29.4)	(28.9 – 29.2)	(28.9 – 29.2)	(29.1 – 29.4)	(29.1 – 29.3)	(28.9 – 29.2)	(29.0 – 29.2)
	50	21.5	21.2	21	20.6	20.5	20.4	20.5	20.5	20.3	20.4
		(21.4 – 21.7)	(21.0 – 21.3)	(20.9 – 21.1)	(20.5 – 20.8)	(20.4 – 20.6)	(20.2 – 20.5)	(20.4 – 20.6)	(20.4 – 20.6)	(20.2 – 20.4)	(20.3 – 20.5)
	60	14.4	14.1	13.9	13.5	13.5	13.3	13.4	13.3	13.2	13.1
		(14.3 – 14.6)	(14.0 – 14.2)	(13.8 – 14.0)	(13.4 – 13.7)	(13.4 – 13.6)	(13.2 – 13.4)	(13.3 – 13.5)	(13.2 – 13.4)	(13.1 – 13.3)	(13.0 – 13.2)
	70	9.0	8.8	8.7	8.5	8.3	8.2	8.3	8.2	8.0	8.1
		(8.9 – 9.1)	(8.7 – 8.9)	(8.6 – 8.8)	(8.4 – 8.6)	(8.3 – 8.4)	(8.1 – 8.3)	(8.2 – 8.4)	(8.1 – 8.3)	(8.0 – 8.1)	(8.0 – 8.2)
	80	5.1	5.0	5.1	4.8	4.7	4.7	4.7	4.6	4.4	4.5
		(5.0 – 5.2)	(4.9 – 5.1)	(5.0 – 5.2)	(4.7 – 4.9)	(4.6 – 4.8)	(4.6 – 4.8)	(4.6 – 4.8)	(4.5 – 4.7)	(4.4 – 4.5)	(4.5 – 4.6)
Women	20	55.1	54.7	54.4	54.0	53.7	53.6	53.8	53.6	53.4	53.4
		(54.9 – 55.3)	(54.6 – 54.9)	(54.3 – 54.6)	(53.8 – 54.1)	(53.6 – 53.9)	(53.4 – 53.7)	(53.6 – 53.9)	(53.5 – 53.8)	(53.2 – 53.5)	(53.3 – 53.6)
	30	45.3	44.9	44.7	44.2	44.0	43.8	44.0	43.8	43.6	43.7
		(45.2 – 45.5)	(44.8 – 45.1)	(44.5 – 44.8)	(44.1 – 44.4)	(43.8 – 44.1)	(43.7 – 44.0)	(43.8 – 44.1)	(43.7 – 44.0)	(43.5 – 43.8)	(43.5 – 43.8)
	40	35.7	35.3	35.1	34.7	34.5	34.3	34.4	34.3	34.1	34.1
		(35.5 – 35.8)	(35.1 – 35.4)	(34.9 – 35.2)	(34.6 – 34.8)	(34.3 – 34.6)	(34.2 – 34.4)	(34.3 – 34.6)	(34.2 – 34.4)	(34.0 – 34.3)	(34.0 – 34.3)
	50	26.6	26.2	26.0	25.7	25.4	25.2	25.4	25.2	25.1	25.1
		(26.5 – 26.7)	(26.1 – 26.4)	(25.9 – 26.1)	(25.5 – 25.8)	(25.3 – 25.6)	(25.1 – 25.3)	(25.3 – 25.5)	(25.1 – 25.4)	(25.0 – 25.2)	(25.0 – 25.2)
	60	18.5	18.2	18.0	17.7	17.5	17.3	17.4	17.3	17.2	17.2
		(18.4 – 18.6)	(18.1 – 18.4)	(17.9 – 18.1)	(17.6 – 17.8)	(17.4 – 17.6)	(17.2 – 17.4)	(17.3 – 17.5)	(17.2 – 17.4)	(17.1 – 17.3)	(17.1 – 17.3)
	70	11.6	11.4	11.3	11.1	11.0	10.8	11.0	10.9	10.8	10.9
		(11.5 – 11.7)	(11.3 – 11.5)	(11.2 – 11.4)	(11.0 – 11.2)	(10.9 – 11.0)	(10.7 – 10.9)	(10.9 – 11.1)	(10.8 – 10.9)	(10.7 – 10.9)	(10.8 – 11.0)
	80	6.2	6.2	6.1	5.9	5.9	5.8	6.0	5.8	5.7	5.9
		(6.2 – 6.3)	(6.1 – 6.2)	(6.1 – 6.2)	(5.8 – 6.0)	(5.8 – 5.9)	(5.7 – 5.8)	(5.9 – 6.0)	(5.8 – 5.9)	(5.6 – 5.7)	(5.8 – 5.9)

### Life expectancy with type 2 diabetes

Life expectancy with type 2 diabetes increased steadily over the observation period from 2005 to 2014 (Table 4). In agreement with the previous findings, life expectancy with type 2 diabetes was higher in women. The development of life expectancies with type 2 diabetes was almost similar for ages 20 to 40, although changes could be observed from the age of 50 onwards. For instance, men at the age of 50 were expected to spend 5.4 years of their remaining total life expectancy with type 2 diabetes in 2005 and 7.6 years in 2014. Women at age 50 had a life expectancy with type 2 diabetes of 6.3 years in 2005 and 8.3 years in 2014. For all ages and both sexes, the increases when comparing 2014 to 2005 were significant.

**Table 4:** Life expectancy with type 2 diabetes and confidence intervals (95%) by year and sex at selected ages

	Age	2005	2006	2007	2008	2009	2010	2011	2012	2013	2014
Men	20	5.7	6.2	6.5	6.9	7.1	7.5	7.6	7.8	7.7	8.0
		(5.5 – 5.8)	(6.0 – 6.4)	(6.4 – 6.7)	(6.7 – 7.0)	(6.9 – 7.3)	(7.4 – 7.7)	(7.5 – 7.8)	(7.6 – 7.9)	(7.6 – 7.9)	(7.9 – 8.2)
	30	5.7	6.2	6.6	6.9	7.1	7.6	7.6	7.8	7.7	8.1
		(5.5 – 5.9)	(6.1 – 6.4)	(6.4 – 6.7)	(6.7 – 7.1)	(7.0 – 7.3)	(7.4 – 7.7)	(7.5 – 7.8)	(7.6 – 7.9)	(7.6 – 7.9)	(7.9 – 8.2)
	40	5.7	6.2	6.5	6.8	7.1	7.5	7.6	7.7	7.6	8.0
		(5.5 – 5.8)	(6.0 – 6.3)	(6.4 – 6.7)	(6.7 – 7.0)	(6.9 – 7.2)	(7.3 – 7.6)	(7.4 – 7.7)	(7.6 – 7.8)	(7.5 – 7.8)	(7.8 – 8.1)
	50	5.4	5.9	6.3	6.5	6.8	7.2	7.2	7.3	7.3	7.6
		(5.3 – 5.6)	(5.8 – 6.1)	(6.2 – 6.4)	(6.4 – 6.7)	(6.6 – 6.9)	(7.0 – 7.3)	(7.1 – 7.3)	(7.2 – 7.5)	(7.2 – 7.4)	(7.5 – 7.7)
	60	4.7	5.2	5.5	5.7	6.0	6.3	6.4	6.5	6.5	6.7
		(4.6 – 4.8)	(5.0 – 5.3)	(5.3 – 5.6)	(5.6 – 5.8)	(5.9 – 6.1)	(6.2 – 6.4)	(6.3 – 6.5)	(6.4 – 6.6)	(6.4 – 6.6)	(6.6 – 6.8)
	70	3.4	3.7	4.0	4.2	4.4	4.7	4.8	4.8	4.8	5.1
		(3.3 – 3.5)	(3.6 – 3.8)	(3.9 – 4.1)	(4.1 – 4.3)	(4.3 – 4.5)	(4.6 – 4.7)	(4.7 – 4.9)	(4.8 – 4.9)	(4.8 – 4.9)	(5.0 – 5.2)
	80	1.9	2.1	2.3	2.4	2.5	2.7	2.8	2.8	2.9	3.1
		(1.8 – 2.0)	(2.0 – 2.2)	(2.2 – 2.4)	(2.3 – 2.5)	(2.4 – 2.6)	(2.6 – 2.8)	(2.7 – 2.9)	(2.8 – 2.9)	(2.8 – 2.9)	(3.0 – 3.1)
Women	20	6.6	7.1	7.5	7.8	8.0	8.4	8.5	8.7	8.6	9.0
		(6.4 – 6.7)	(7.0 – 7.3)	(7.3 – 7.7)	(7.7 – 8.0)	(7.9 – 8.2)	(8.2 – 8.5)	(8.4 – 8.7)	(8.5 – 8.8)	(8.4 – 8.7)	(8.9 – 9.2)
	30	6.5	7.1	7.5	7.8	8.0	8.3	8.5	8.6	8.5	8.9
		(6.4 – 6.7)	(6.9 – 7.2)	(7.3 – 7.6)	(7.6 – 7.9)	(7.8 – 8.1)	(8.2 – 8.5)	(8.3 – 8.6)	(8.5 – 8.7)	(8.4 – 8.7)	(8.8 – 9.1)
	40	6.5	7.0	7.3	7.6	7.8	8.2	8.3	8.4	8.3	8.7
		(6.3 – 6.6)	(6.8 – 7.1)	(7.2 – 7.5)	(7.5 – 7.8)	(7.7 – 8.0)	(8.0 – 8.3)	(8.2 – 8.4)	(8.3 – 8.5)	(8.2 – 8.5)	(8.6 – 8.8)
	50	6.3	6.7	7.1	7.3	7.5	7.8	7.9	8.0	8.0	8.3
		(6.1 – 6.4)	(6.6 – 6.9)	(6.9 – 7.2)	(7.2 – 7.5)	(7.4 – 7.6)	(7.7 – 7.9)	(7.8 – 8.1)	(7.9 – 8.2)	(7.9 – 8.1)	(8.2 – 8.4)
	60	5.6	6.0	6.3	6.5	6.7	7.0	7.1	7.2	7.2	7.5
		(5.5 – 5.7)	(5.9 – 6.1)	(6.2 – 6.4)	(6.4 – 6.6)	(6.6 – 6.8)	(6.9 – 7.1)	(7.0 – 7.2)	(7.1 – 7.3)	(7.1 – 7.3)	(7.4 – 7.6)
	70	4.3	4.6	4.9	5.1	5.2	5.4	5.5	5.6	5.6	5.8
		(4.2 – 4.4)	(4.6 – 4.7)	(4.8 – 4.9)	(5.0 – 5.1)	(5.1 – 5.3)	(5.3 – 5.5)	(5.4 – 5.6)	(5.5 – 5.7)	(5.5 – 5.6)	(5.7 – 5.9)
	80	2.6	2.8	2.9	3.0	3.1	3.2	3.3	3.4	3.4	3.6
		(2.5 – 2.6)	(2.7 – 2.8)	(2.9 – 3.0)	(2.9 – 3.1)	(3.1 – 3.2)	(3.2 – 3.3)	(3.3 – 3.4)	(3.3 – 3.4)	(3.3 – 3.4)	(3.6 – 3.7)

### Proportion of life expectancy without diabetes to total life expectancy

Overall, a continuous decline in the proportion of life expectancy without diabetes to total life expectancy was visible in both sexes with increasing age but also over the 10-year period (Table 5). In contrast to the aforementioned findings, the relative examination of years without type 2 diabetes and total life expectancy revealed a similar development for men and women, because the figures were not substantially different. This means that the relative amount of years without type 2 diabetes to total life expectancy was almost the same for men and women at every selected age or year. Stronger decreases over the observed time period were visible in the older age groups after the age of 50. At the age of 20, the proportion of life expectancy without diabetes to total life expectancy of men was 0.90 in 2005 and dropped slightly to 0.86 in 2014, whereas in women, it was 0.89 in 2005 and 0.86 in 2014. In both sexes, the proportion of life expectancy without diabetes to total life expectancy amounted to 0.73 for age 70 in 2005 and decreased to 0.61 in men and 0.65 in women in 2014. When comparing 2014 to 2005, the decreases were significant for all ages and both sexes.

**Table 5:** Proportion of life expectancy without diabetes to total life expectancy and confidence intervals (95%) by year and sex at selected ages

	Age	2005	2006	2007	2008	2009	2010	2011	2012	2013	2014
Men	20	0.90	0.89	0.88	0.88	0.87	0.86	0.86	0.86	0.86	0.86
		(0.89 – 0.90)	(0.88 – 0.89)	(0.88 – 0.88)	(0.87 – 0.88)	(0.87 – 0.87)	(0.86 – 0.87)	(0.86 – 0.87)	(0.86 – 0.86)	(0.86 – 0.86)	(0.85 – 0.86)
	30	0.87	0.86	0.86	0.85	0.84	0.84	0.84	0.83	0.83	0.83
		(0.87 – 0.88)	(0.86 – 0.87)	(0.85 – 0.86)	(0.85 – 0.85)	(0.84 – 0.85)	(0.83 – 0.84)	(0.83 – 0.84)	(0.83 – 0.84)	(0.83 – 0.84)	(0.82 – 0.83)
	40	0.84	0.83	0.82	0.81	0.80	0.80	0.79	0.79	0.79	0.79
		(0.84 – 0.85)	(0.82 – 0.83)	(0.82 – 0.82)	(0.81 – 0.81)	(0.80 – 0.81)	(0.79 – 0.80)	(0.79 – 0.80)	(0.79 – 0.80)	(0.79 – 0.80)	(0.78 – 0.79)
	50	0.80	0.78	0.77	0.76	0.75	0.74	0.74	0.74	0.74	0.73
		(0.79 – 0.80)	(0.78 – 0.79)	(0.76 – 0.77)	(0.75 – 0.76)	(0.75 – 0.76)	(0.74 – 0.74)	(0.74 – 0.74)	(0.73 – 0.74)	(0.73 – 0.74)	(0.72 – 0.73)
	60	0.75	0.73	0.72	0.70	0.69	0.68	0.68	0.67	0.67	0.66
		(0.75 – 0.76)	(0.73 – 0.74)	(0.71 – 0.72)	(0.70 – 0.71)	(0.69 – 0.70)	(0.67 – 0.68)	(0.67 – 0.68)	(0.67 – 0.68)	(0.66 – 0.67)	(0.66 – 0.67)
	70	0.73	0.70	0.69	0.67	0.66	0.64	0.64	0.63	0.62	0.61
		(0.72 – 0.73)	(0.69 – 0.71)	(0.68 – 0.69)	(0.66 – 0.68)	(0.65 – 0.66)	(0.63 – 0.64)	(0.63 – 0.64)	(0.62 – 0.63)	(0.62 – 0.63)	(0.61 – 0.62)
	80	0.73	0.70	0.69	0.67	0.65	0.64	0.63	0.62	0.61	0.60
		(0.71 – 0.74)	(0.69 – 0.72)	(0.67 – 0.70)	(0.66 – 0.68)	(0.64 – 0.67)	(0.63 – 0.65)	(0.62 – 0.64)	(0.61 – 0.63)	(0.60 – 0.62)	(0.59 – 0.61)
Women	20	0.89	0.88	0.88	0.87	0.87	0.86	0.86	0.86	0.86	0.86
		(0.89 – 0.90)	(0.88 – 0.89)	(0.88 – 0.88)	(0.87 – 0.88)	(0.87 – 0.87)	(0.86 – 0.87)	(0.86 – 0.87)	(0.86 – 0.86)	(0.86 – 0.86)	(0.85 – 0.86)
	30	0.87	0.86	0.86	0.85	0.85	0.84	0.84	0.84	0.84	0.83
		(0.87 – 0.88)	(0.86 – 0.87)	(0.85 – 0.86)	(0.85 – 0.85)	(0.84 – 0.85)	(0.84 – 0.84)	(0.84 – 0.84)	(0.83 – 0.84)	(0.83 – 0.84)	(0.83 – 0.83)
	40	0.85	0.84	0.83	0.82	0.81	0.81	0.81	0.80	0.80	0.80
		(0.84 – 0.85)	(0.83 – 0.84)	(0.82 – 0.83)	(0.82 – 0.82)	(0.81 – 0.82)	(0.80 – 0.81)	(0.80 – 0.81)	(0.80 – 0.81)	(0.80 – 0.81)	(0.79 – 0.80)
	50	0.81	0.80	0.79	0.78	0.77	0.76	0.76	0.76	0.76	0.75
		(0.81 – 0.81)	(0.79 – 0.80)	(0.78 – 0.79)	(0.77 – 0.78)	(0.77 – 0.78)	(0.76 – 0.77)	(0.76 – 0.77)	(0.75 – 0.76)	(0.76 – 0.76)	(0.75 – 0.76)
	60	0.77	0.75	0.74	0.73	0.72	0.71	0.71	0.71	0.71	0.70
		(0.76 – 0.77)	(0.75 – 0.76)	(0.74 – 0.75)	(0.73 – 0.73)	(0.72 – 0.73)	(0.71 – 0.72)	(0.71 – 0.71)	(0.70 – 0.71)	(0.70 – 0.71)	(0.69 – 0.70)
	70	0.73	0.71	0.70	0.69	0.68	0.67	0.67	0.66	0.66	0.65
		(0.72 – 0.73)	(0.71 – 0.72)	(0.69 – 0.70)	(0.68 – 0.69)	(0.67 – 0.68)	(0.66 – 0.67)	(0.66 – 0.67)	(0.66 – 0.66)	(0.66 – 0.66)	(0.65 – 0.66)
	80	0.71	0.69	0.68	0.66	0.65	0.64	0.64	0.63	0.63	0.62
		(0.70 – 0.72)	(0.68 – 0.70)	(0.67 – 0.68)	(0.66 – 0.67)	(0.65 – 0.66)	(0.64 – 0.65)	(0.63 – 0.65)	(0.63 – 0.64)	(0.62 – 0.64)	(0.61 – 0.63)

## Discussion

The objective of our study was to examine life expectancy without type 2 diabetes and life expectancy with type 2 diabetes. We used German administrative claims data and examined the development of different life expectancies over a period of 10 years. The total life expectancy was noted to be increasing slightly. While this finding applies more to men than to women, it should be kept in mind that the female total life expectancy of the population under study was already higher than in males. The reduction of life expectancy without type 2 diabetes was more pronounced in women than in men. However, life expectancy with type 2 diabetes increased in both, and the proportion of life expectancy free of diabetes to total life expectancy decreased in men and women, thus indicating that the relative amount of type 2 diabetes free life years decreased over the observation period. Therefore, our hypothesis that life expectancy with type 2 diabetes increases was confirmed for both sexes, and it can be concluded that an expansion of morbidity has occurred.

The increase in age-standardized prevalence rates as well as in life expectancy with type 2 diabetes is in accordance with reported increases in prevalence and with the rising number of younger patients with type 2 diabetes [8–10]. High obesity rates in German children and young adults might play an important role in the early development of the disease [25, 26]. Lifestyle changes, especially of diet and exercise, have been demonstrated to be influential on the long-term development of type 2 diabetes [27]. Our study illustrates the importance of preventive action towards reducing obesity, especially in childhood, in order to avoid type 2 diabetes-related complications and premature mortality.

The relative amount of life years without type 2 diabetes decreased, as indicated by the proportion of life expectancy without diabetes to total life expectancy, thus raising concern about the future development. Our results are in line with projections for Australia and Mexico that indicate an increase in diabetes prevalence and lifetime risk of developing the disease [28, 29]. If this trend persists, it can be assumed that disease-related complications and health care costs will increase accordingly. For example, Wong et al. [30] discovered higher complication and mortality rates in patients with a young onset age of type 2 diabetes. Bardenheier et al. [31] found higher disability and mortality rates in men and women with diabetes than in the group without the disease. Based on a prediction for Germany, the health costs of type 2 diabetes are estimated to increase by 79% from 11.8 billion euro in 2010 to 21.1 billion euro in 2040 if the therapeutic procedures remain unchanged [32]. Furthermore, the slight decrease in mortality found in our data could also be responsible for the increase in life expectancy with type 2 diabetes. This finding is in line with previous studies from Denmark [33] and the United States [34] that show a higher lifetime risk of type 2 diabetes due to increasing diabetes incidence and declining mortality.

It can be discussed whether earlier onset of type 2 diabetes may be due to improved diagnostics and earlier detection, or whether onset has actually shifted towards younger age groups. In both cases, a population would appear increasingly morbid. As diagnostic procedures have not changed since disease management programs (DMP) were introduced in 2002 in Germany [17], the observed changes might be attributed to an earlier onset age. It is also conceivable that earlier detection and better therapies might prevent late complications and thus improve the quality of life of patients. Accordingly, disease courses may be less severe, and health costs may decrease. To decide between these alternatives, future studies should also include subjective information on the quality of life of patients with type 2 diabetes. Then, a distinction between a mere expansion of morbidity and the dynamic equilibrium as an alternative pattern would be possible. Even in the case of prolonged morbidity periods, improvements of quality of life in type 2 diabetes patients would point towards a dynamic equilibrium. Instead of quality of life measures as an indicator of disease severity, one might also consider the occurrence of diabetes-related complications, especially when using claims data sources.

The observation period from 2005 to 2014 may be viewed as rather short, but it has to be kept in mind that changes in the development of morbidity and mortality are constant and consistently moving in the same direction. The advantages of the claims dataset are the broad age range and the large number of individuals, which made it possible to apply period life tables. Furthermore, an advantage of the administrative nature of health insurance data is the absence of selection and response bias. For all insured individuals, complete health care information is available, even for those with deteriorating health status who are more likely to be non-respondents in surveys [35, 36]. The distributions of age, gender, and employment rates in our data were similar to the population of Lower Saxony and Germany. However, the qualification level of the insurance population was lower [37].

## Conclusions

We found an increase in total life expectancy but also of life expectancy with type 2 diabetes between 2005 and 2014. These findings indicate an expansion of morbidity. This finding highlights the importance of preventive measures to avoid the deterioration of population heath in terms of more disease-related complications and increasing health care costs. However, possible improvements of therapies and quality of life in type 2 diabetes patients should be subject to future analyses in order to determine whether a dynamic equilibrium is present rather than a morbidity expansion.
